# Medicaid Expansion and 30‐Day Mortality After Heart Failure Hospitalization: A Nationwide Study

**DOI:** 10.1002/clc.70240

**Published:** 2025-12-20

**Authors:** Julianne Ghiorzi, Julie D. Sill, Rehan Qayyum

**Affiliations:** ^1^ Department of Medicine, Eastern Virginia Medical School at Old Dominion University Student Research Associate Virginia USA; ^2^ Department of Medicine, Eastern Virginia Medical School at Old Dominion University Research Manager Virginia USA; ^3^ Department of Medicine Eastern Virginia Medical School at Old Dominion University Virginia USA

**Keywords:** 30‐day post‐discharge mortality, affordable care act, heart failure, Medicaid, mortality

## Abstract

**Aims:**

Medicaid Expansion (ME) under the Affordable Care Act sought to improve health access though not all states expanded Medicaid. Our goal is to examine whether ME impacts 30‐day post‐discharge mortality rates for heart failure (HF) hospitalizations.

**Methods:**

We constructed a data set incorporating 30‐day HF mortality and hospital service area (HSA) characteristics using five sources: (1) Centers for Medicare and Medicaid Services, (2) Medicaid Budget and Expenditure System, (3) US Census Bureau, (4) Dartmouth Atlas of Healthcare, (5) Kaiser Family Foundation. We categorized states as expanding Medicaid by 2014 or not expanding until 2020, excluding five states that expanded between 2014 and 2020. A difference‐in‐difference (DID) model, adjusted for hospital and HSA factors, was used for analysis.

**Results:**

Among 3839 hospitals, 52% were in ME states. Before 2014, 30‐day mortality rates were higher in non‐ME state hospitals than in ME state hospitals (11.6% vs. 11.4%; *p* < 0.001). After 2014, rates increased in non‐ME state hospitals (change = 0.11%, 95% CI: 0.04% to 0.18%) but remained unchanged in ME state hospitals (change = 0.01%, 95% CI: –0.07% to 0.08%). The adjusted DID analysis showed a significant disparity in trends between ME and non‐ME states (adjusted DID: −0.11%, 95% CI: –0.21% to –0.02%; *p* = 0.02). A dose‐response relationship revealed that each increase of 10,000 new Medicaid enrollees was associated with 0.002% (95% CI: –0.003 to –0.001; *p* < 0.001) reduced 30‐day HF mortality.

**Conclusions:**

Hospitals in ME states maintained stable mortality rates, contrasting with increases in non‐ME states, suggesting that improved healthcare access through ME contributed to better outcomes.

## Graphical abstract

This study examines the impact of Medicaid Expansion on 30‐day post‐discharge mortality following heart failure hospitalization. Using an adjusted national data set, we found mortality rose in non‐expansion states but remained stable in expansion states. Increased Medicaid enrollment correlated with reduced mortality, suggesting improved outcomes through expanded healthcare access.

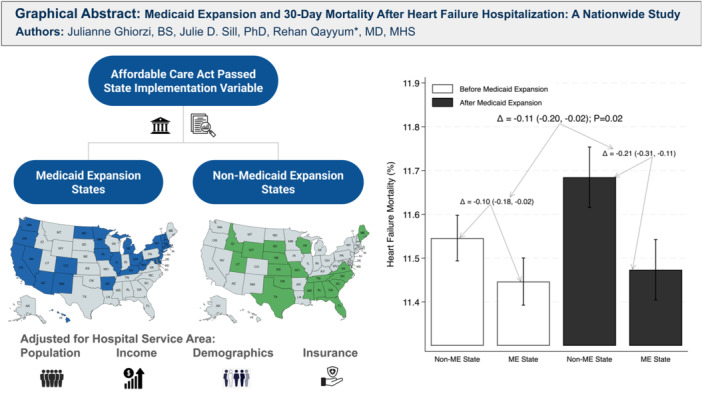

## 1 Introduction

The Affordable Care Act (ACA), enacted in 2010, aimed to increase health insurance accessibility and expand Medicaid to cover adults aged 19–64 with incomes up to 138% of the federal poverty level [1, 2]. This Medicaid Expansion (ME) significantly improved healthcare access for low‐income adults, reducing health disparities [3]. However, a 2012 Supreme Court decision allowed individual states to decide whether to adopt the expansion, resulting in varied implementation across the United States [4]. States that adopted ME saw better healthcare access and follow‐up care for previously uninsured patients, while non‐ME states saw no change [5]. This uneven adoption created a natural experiment offering an opportunity to examine the policy's effects on population health and healthcare equity.

Previous studies have assessed the impact of ME on various health outcomes, showing notable reductions in mortality rates for breast, colon, and lung cancers, as well as a decline in all‐cause mortality and hospital readmissions following expansion [6, 7, 8]. Cardiovascular mortality trends have also been analyzed, with findings indicating a slower rise in cardiovascular mortality in states with ME compared to non‐ME states [9]. However, the effect of ME on heart failure (HF) mortality remains understudied. ME has not been linked to increased access to left ventricular assist devices in HF patients, nor has it reduced in‐hospital cardiovascular mortality [10, 11]. A study specifically investigating HF mortality rates before and after ME implementation similarly found no significant change [12]. This study, however, relied on data from a voluntary hospital database committed to quality improvement, which limits the generalizability of its findings. Additionally, it focused solely on in‐hospital mortality, excluding post‐discharge outcomes that could provide valuable insights into both acute care and continuity of care. Therefore, a significant gap remains in national studies exploring the effect of ME on 30‐day post‐discharge HF mortality.

To address this gap in the literature, we analyzed nationwide data from the Centers for Medicare and Medicaid Services (CMS) on HF mortality before and after ME. Since 2007, CMS has reported a 3‐year running average of 30‐day risk‐standardized mortality rates for Medicare patients with HF discharged from acute care hospitals across the country [13]. Using this national data set and focusing on 30‐day post‐discharge mortality as the primary outcome, we aimed to assess the effect of ME on post‐discharge continuity of care and short‐term outcomes in HF patients. We hypothesized that states adopting ME would experience a reduction in 30‐day HF mortality post‐hospitalization compared to states with delayed or no adoption and that states with higher number of new Medicaid enrollees after ME, will have larger reductions in 30‐day HF mortality.

## 2 Methods

This study utilized five data sets to comprehensively evaluate the impact of ME. The first data set, obtained from the CMS, provided information on hospital characteristics and risk‐standardized 30‐day HF mortality rates. The CMS‐reported 30‐day mortality rates were based on a 3‐year rolling average. For this study, we used data spanning five 3‐year cycles, covering a 15‐year period from July 2005 to December 2019. Data after 2019 were excluded to avoid confounding effects from the COVID‐19 pandemic, which disrupted healthcare delivery and could influence the relationship between ME and mortality outcomes.

To distinguish new Medicaid enrollments resulting specifically from ME from the overall Medicaid population, a second data set from the Medicaid Budget and Expenditure System (MBES) was incorporated [14]. For states that adopted ME, the data set specifies the number of individuals enrolled under the new adult eligibility category, referred to as the “VIII Group.” This data, covering all 50 states, the District of Columbia, and U.S. territories, enabled the distinction between newly eligible and previously eligible Medicaid enrollees by state.

**Figure 1:**
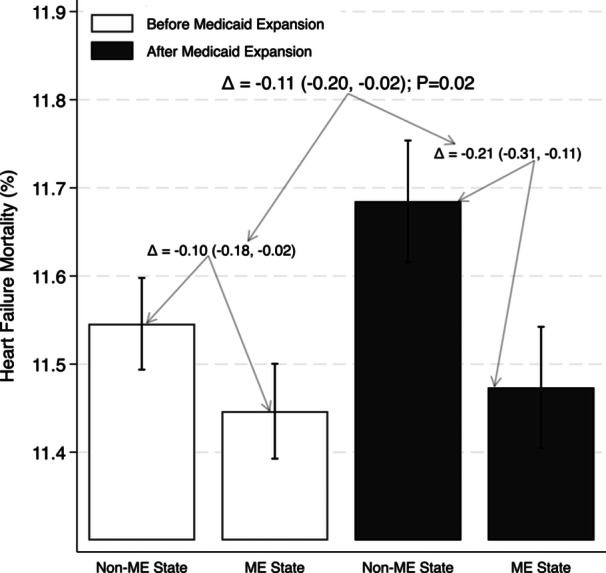
Heart failure mortality rates before and after medicaid expansion (ME). This figure displays the before and after comparison of 30‐day risk‐standardized heart failure mortality rates for hospitals located in ME States and non‐ME States. Results were adjusted for hospital ownership, teaching status, number of hospital beds, number of nurses, rural versus urban hospital location, dual‐eligible Medicaid–Medicare enrollees in the state, and characteristics of the hospital service area population (total population, median income, poverty percentage, African American percentage, and percentage with insurance). The delta signifies the difference between two measures of interest.

The third data set, obtained from the Kaiser Family Foundation, supplied detailed timelines of ME adoption by state. For analysis, states were grouped into two categories: (1) “ME States,” comprising 27 states and the District of Columbia that expanded Medicaid in 2014, and (2) “Non‐ME States,” consisting of 18 states that delayed expansion until at least 2019. Five states that expanded Medicaid between 2014 and 2019 were excluded to maintain consistency in the comparison groups (Supporting Information S1: Figure 1).

The fourth data set, obtained from the US Census Bureau, provided regional demographic and economic data. The fifth data set, derived from the Dartmouth Atlas of Healthcare, included crosswalk files defining 3436 hospital service areas (HSAs). HSAs are delineated by assigning ZIP codes to hospital catchment areas where the largest number of Medicare beneficiaries receive care [15]. To integrate US Census Bureau data with HSAs, we utilized these crosswalk files, combining ZIP code‐level data with HSA characteristics. This approach allowed us to construct a comprehensive data set that incorporated hospital features, 30‐day HF mortality, and HSA‐level demographic information.

## 3 Statistical Methods

Data were summarized using means (SD) or percentages, and differences between variables were assessed using the Chi‐square test or the Wilcoxon rank‐sum test, as appropriate. To evaluate the relationship between ME and 30‐day HF mortality, we employed a difference‐in‐difference (DID) framework using a mixed linear model to account for repeated observations from the same hospital; hospitals served as the unit of analysis. These models allowed for the assessment of changes in HF mortality with ME while considering correlations between repeated measurements. We tested the parallel‐trend assumption for the DID model graphically as well as using formal statistical testing.

Fully adjusted models accounted for potential time‐invariant confounders, including hospital ownership, teaching status, number of beds, nurse staffing levels, rural versus urban location, the proportion of dual Medicaid–Medicare enrollees in the state, and HSA characteristics such as total population, median income, poverty rate, percentage of African American residents, and insurance coverage rates.

To validate the relationship between ME and 30‐day HF mortality, we examined whether a dose‐response relationship existed between the number of “VIII group” enrollees and 30‐day HF mortality in ME states, with and without adjusting for potential confounders. Statistical significance was set at *p* < 0.05. Linear mixed models were specified using the ‘mixed’ command in Stata 18.1 (StataCorp LLC) with unstructured covariance matrices, and standard errors were estimated using the robust sandwich estimator.

## 4 Results

The study included 3839 hospitals, with 2012 (52.4%) located in ME states and 1827 (47.6%) in non‐ME states. Hospitals in non‐ME states were more likely to be for‐profit (21.4% vs. 10.0%; *p* < 0.001), rural (47.4% vs. 35.8%; *p* < 0.001), and nonteaching (83.7% vs. 69.9%; *p* < 0.001) compared to those in ME states. Non‐ME state hospitals also had fewer employed nurses (177 vs. 223; *p* < 0.001) and fewer hospital beds (175 vs. 209; *p* < 0.001). There was no difference in the number of dual Medicare‐Medicaid enrollees between the ME and non‐ME states. The HSA in non‐ME states had a higher proportion of African Americans (13.5% vs. 7.18%; *p* < 0.001), higher poverty rates (16.9% vs. 15.0%; *p* < 0.001), lower mean income levels ($46 500 vs. $55 600; *p* < 0.001), and a lower percentage of insured patients (82.5% vs. 85.9%; *p* < 0.001) compared to ME states (Table 1).

**Table 1:** Baseline hospital and hospital service area characteristics‐ The table displays the baseline hospital service area characteristics for both Medicaid Expansion (ME) and non‐ME states, as well as if the differences in characteristics are statistically significant.

Variables	Non‐ME states (*N* = 1827; 47.6%)	ME states (*N* = 2012; 52.4%)	*p* value*
*Hospital characteristics*			
HF mortality, %	11.62 (1.43)	11.44 (1.59)	< 0.0001
*Ownership*			
Government, *n* (%)	497 (27.2)	381 (18.9)	< 0.0001
Non‐profit, *n* (%)	939 (51.4)	1430 (71.1)	
For‐profit, *n* (%)	391 (21.4)	201 (10.0)	
Rural Location, *n* (%)	867 (47.4)	721 (35.8)	< 0.0001
Teaching hospital, *n* (%)	297 (16.3)	606 (30.1)	< 0.0001
Nurses Employed by Hospital, *n* (%)	177 (332)	223 (299)	< 0.0001
Beds in hospital	175 (217)	209 (213)	< 0.0001
*HSA characteristics*			
Total population in HSA (in 10 000 s)	30.1 (62.3)	32.1 (55.0)	< 0.0001
Median age	40.0 (4.8)	40.5 (4.9)	0.20
African American in HSA, %	13.5 (15.1)	7.18 (6.70)	< 0.0001
Mean income in HSA (in $10 000 s)	4.65 (1.27)	5.56 (1.82)	< 0.0001
Poverty in HSA, %	16.9 (6.21)	15.0 (6.67)	< 0.0001
Insurance in HSA, %	82.5 (6.09)	85.9 (6.26)	< 0.0001
*State characteristics*			
State dual (Medicaid‐Medicare) enrollees (in 10 000 s)	19.4 (19.8)	19.9 (27.4)	0.77
New Medicaid to total Medicaid enrollees ratio (10 000 s)	—	58.6 (81.5)	NA

*Note:* *p values based on chi‐square test or Wilcoxon rank‐sum test.

Abbreviations: HF = Heart Failure, HSA = Hospital Service Area.

Before ME in 2014, the 30‐day HF mortality rate was significantly higher in hospitals located in non‐ME states compared to ME states (11.62% vs. 11.44%; *p* < 0.0001). After 2014, the 30‐day HF mortality rate increased to 11.73% in non‐ME states (change = 0.11%; *p* = 0.001) while remaining relatively unchanged at 11.43% in ME states (change = 0.01%; *p* = 0.84). Notably, the difference in mortality rates between ME and non‐ME states before and after ME was statistically significant (DID = −0.11%; 95% CI: −0.20%, −0.02%; *p* = 0.02). This finding remained significant even after adjusting for hospital‐level, HSA‐level, and state‐level confounders (DID = −0.11%; 95% CI: −0.20%, −0.02%; *p* = 0.02) (Figure 1 and Supporting Information S1: Tables 1 and 2).

**Figure 2:**
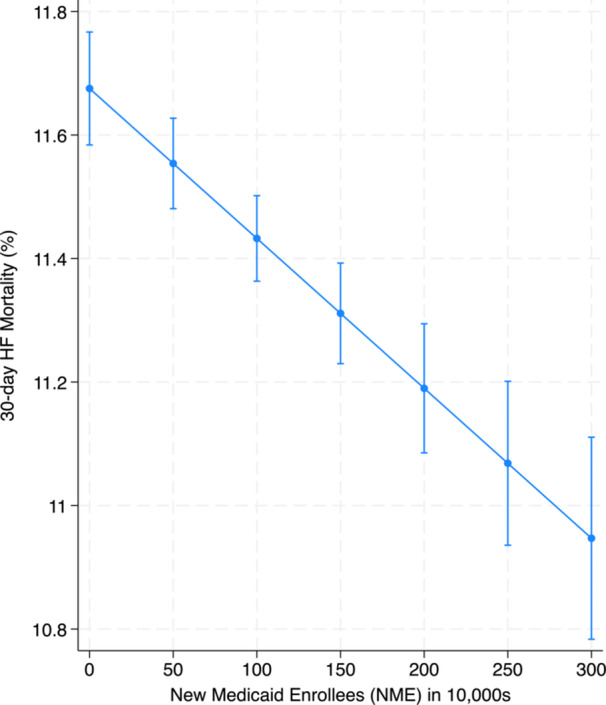
Effect of new medicaid enrollees on heart failure (HF) mortality. This figure displays the relationship between 30‐day risk‐standardized HF mortality and the number of new Medicaid enrollees (in 10 000 s) in the Medicaid Expansion (ME) states after Medicaid expansion. Results were adjusted for hospital ownership, teaching status, number of hospital beds, number of nurses, rural versus urban hospital location, dual‐eligible Medicaid–Medicare enrollees in the state, and characteristics of the hospital service area population (total population, median income, poverty percentage, African American percentage, and percentage with insurance).

In assessing a dose‐response relationship, we observed that an increase in the number of new Medicaid enrollees was significantly associated with a reduction in 30‐day HF mortality (–0.003% per 10 000 new enrollees; 95% CI: –0.004, –0.002; *p* < 0.001). This association remained robust after adjusting for potential confounders (−0.002% per 10 000 new enrollees; 95% CI: −0.003, −0.001; *p* < 0.001) (Figure 2 and Supporting Information S1: Table 3).

## 5 Discussion

In this study, we found states that adopted ME experienced a significant reduction in 30‐day HF mortality following hospitalization compared to states without ME adoption. The reduction in HF mortality was robust to potential confounders at the hospital, HSA, and State levels. Furthermore, states with a higher number of new Medicaid enrollees after ME implementation demonstrated larger reductions in 30‐day HF mortality, confirming a dose‐response relationship between Medicaid enrollment and improved outcomes. Although the absolute effect size was modest, the persistence of this association after adjustment for confounders and the observed dose‐dependence support its relevance at the population level.

Several studies have demonstrated a decrease in mortality associated with ME across various patient populations. This survival benefit has been observed in patients with breast, gastric, cervical, and lung cancer [16, 17, 18], lymphoma [19], and among formerly incarcerated individuals [20]. However, the impact of ME on mortality in patients with cardiovascular diseases has been less consistent. A systematic review found that only two of the six studies reviewed reported a decrease in cardiac mortality [21], while another study found no significant survival benefit or reduction in amputation rates for patients with peripheral arterial disease [22]. Our study adds to this growing body of evidence by demonstrating that ME is associated with reduced 30‐day HF mortality in ME states compared to non‐ME states. Although the observed effect size is modest, it is important to note that HF mortality data include all HF hospitalizations, not solely those of individuals who gained coverage due to ME, which likely dilutes the measurable impact of ME. Moreover, ME is expected to influence a range of health outcomes, including reducing healthcare utilization and hospital readmissions, which collectively contribute to a broader and more substantial improvement in overall health outcomes [8, 23].

ME has been linked to several health benefits through different mechanisms, which likely also contributed to the observed reduction in 30‐day HF mortality following hospitalization. First, the ME has led to increased insurance coverage, especially for low‐income families or uninsured people, improving access to healthcare and the management of cardiac comorbidities such as hypertension and diabetes [21]. These factors are essential in reducing the risk of complications and worsening HF. Additionally, ME has been associated with better adherence to prescribed medications, which directly impacts disease control and patient outcomes [24]. Furthermore, ME has facilitated more timely screenings and diagnoses for conditions like cancer, as well as improved access to cancer‐directed treatments [25]. This pattern of improved healthcare access extends to other areas as well, such as greater access to continuous glucose monitoring in type 1 diabetes, more surgical procedures, and increased kidney transplant opportunities [26, 27, 28]. Although the evidence on health behaviors is mixed, with some studies showing increased HIV screening rates in ME states but no significant change in flu vaccination rates [29], the overall trend suggests that expanded healthcare access leads to earlier diagnosis, timely treatments, and better medication adherence. Lastly, other aspects of the ACA, in addition to ME, may have played a role, including greater access to the private insurance market and subsidies for buying private insurance [3]. These combined factors likely contribute to the reduction in 30‐day HF mortality observed in ME states.

A key strength of our study is its use of a large, nationally representative data set encompassing all US hospitals, providing robust statistical power and generalizability. By integrating data from multiple sources, our analyses are strengthened through adjustments for potential confounders, enabling meaningful comparisons between ME and non‐ME states. Furthermore, the study relies on pre‐pandemic data, thereby avoiding the potential confounding effects of COVID‐19‐related healthcare disruptions. This study is not without limitations. While CMS‐reported 30‐day HF mortality rates are adjusted for patient‐level factors, ensuring valid comparisons across hospitals, one substantial limitation is that the unit of analysis was at the hospital level in this study, which precludes direct inferences about patient‐level factors, such as age, race/ethnicity, and comorbid conditions, all of which may influence 30‐day HF mortality. Another limitation is the reliance on two distinct time points, which raises the possibility of regression to the mean. That said, the data was collected over multiple years within each time period, providing stable and reliable estimates. Additionally, five states expanding Medicaid between 2014 and 2019 were excluded from analysis, improving group comparability but increasing the potential risk of bias due to data omission. Future research employing staggered adoption models to include these states would provide a more nuanced understanding of the policy's effect. As this is an observational study, a definitive cause‐and‐effect relationship cannot be established. The temporal relationship between ME and HF mortality reduction supports the directionality of the relationship from ME to improved HF mortality, but unmeasured factors such as local policy changes, QI initiatives, and differential uptake of the ACCF/AHA Guideline‐Directed Medical Therapy for HF published in 2013 may have confounded observed trends. Similarly, other ACA provisions, such as the establishment of health insurance marketplaces and subsidies, may have contributed to improved outcomes independently of ME, and our study design cannot fully disentangle these effects.

Future research is essential to fully understand the impact of ME on patient health outcomes. However, our study provides important and timely insights based on a national data set. These findings serve as a critical evidence base for policymakers, guiding the development of targeted strategies to enhance patient outcomes and reduce mortality within the US healthcare system. Future studies should explore the underlying mechanisms driving these associations, assess the long‐term effects across diverse patient populations, and evaluate the effectiveness of policy interventions informed by this evidence.

## Funding

The authors received no specific funding for this work.

## Ethics Statement

Study uses publicly available data only and does not require IRB approval.

## Conflicts of Interest

The authors declare no conflicts of interest.

## Supporting information


**Supplementary Figure 1:** Distribution of Medicaid Expansion and non‐Medicaid Expansion States‐ This figure displays a map of the United States of America with states that expanded Medicaid in 2014 labeled in blue and states that did not expand Medicaid until after 2019 labeled in green. States labeled grey were excluded from this study as they expanded Medicaid between 2014 and 2019. **Supplemental Table 1:** Results of mixed linear models with hospital as random intercept and difference in difference framework. The time is divided before and after Medicaid expansion (ME). **Supplementary Table 2:** Results of mixed linear models examining the effect of Medicaid‐expansion on 30‐day heart failure mortality: Hospital was used as random intercept and an interaction between Medicaid‐expansion (ME) and time was included in the model. The time here is divided by non‐overlapping 3‐years running average of 30‐days heart failure (HF) mortality. Two time period (2005‐08 and 2008‐11) were before the ME and rest of the three time periods were after ME. The interaction results below show that the three time periods after ME were associated with lower 30‐day HF mortality in ME states than non‐ME states. **Supplementary Table 3:** Relationship Between Heart Failure Mortality and New Medicaid Enrollees After Medicaid Expansion ‐ This table displays the unadjusted and adjusted relationship between 30‐day risk standardized heart failure mortality and new Medicaid enrollees after Medicaid‐expansion using mixed linear models with hospital as random‐intercepts.

## Data Availability

The data are available publicly.
