# Supplementary material for: Medicaid Expansion and 30‐Day Mortality After Heart Failure Hospitalization: A Nationwide Study
Source: Clin Cardiol. 2025 Dec 20;48(12):e70240. doi: 10.1002/clc.70240 (PMC12717469; doi:10.1002/clc.70240)
Supplement: Supplementary file 1 — Supplementary Figure 1: Distribution of Medicaid Expansion and non‐Medicaid Expansion States‐ This figure displays a map of the United States of America with states that expanded Medicaid in 2014 labeled in blue and states that did not expand Medicaid until after 2019 labeled in green. States labeled grey were excluded from this study as they expanded Medicaid between 2014 and 2019. Supplemental Table 1: Results of mixed linear models with hospital as random intercept and difference in difference framework. The time is divided before and after Medicaid expansion (ME). Supplementary Table 2: Results of mixed linear models examining the effect of Medicaid‐expansion on 30‐day heart failure mortality: Hospital was used as random intercept and an interaction between Medicaid‐expansion (ME) and time was included in the model. The time here is divided by non‐overlapping 3‐years running average of 30‐days heart failure (HF) mortality. Two time period (2005‐08 and 2008‐11) were before the ME and rest of the three time periods were after ME. The interaction results below show that the three time periods after ME were associated with lower 30‐day HF mortality in ME states than non‐ME states. Supplementary Table 3: Relationship Between Heart Failure Mortality and New Medicaid Enrollees After Medicaid Expansion ‐ This table displays the unadjusted and adjusted relationship between 30‐day risk standardized heart failure mortality and new Medicaid enrollees after Medicaid‐expansion using mixed linear models with hospital as random‐intercepts. [file CLC-48-e70240-s001.docx]

**Supplementary Figure 1**: **Distribution of Medicaid Expansion and non-Medicaid Expansion States-** This figure displays a map of the United States of America with states that expanded Medicaid in 2014 labeled in blue and states that did not expand Medicaid until after 2019 labeled in green. States labeled grey were excluded from this study as they expanded Medicaid between 2014 and 2019.


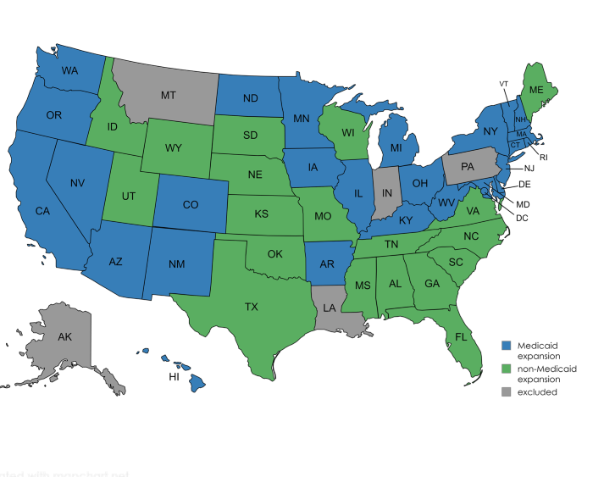


**Supplemental Table 1:** **Results of mixed linear models with hospital as random intercept and difference in difference framework.** The time is divided before and after Medicaid expansion (ME).

|  | **Unadjusted** | **Adjusted** |
| --- | --- | --- |
| Time – Before ME | REF | REF |
| Time – After ME | 0.11 (0.05, 0.18); 0.001 | 0.14 (0.07, 0.21); <0.001 |
| Non-ME State | REF | REF |
| ME State | -0.18 (-0.26, -0.10); <0.0001 | -0.11 (-0.19, -0.03); 0.009 |
| Time-After ME x ME State | -0.11 (-0.20, -0.0); 0.021 | -0.11 (-0.20, -0.02); 0.019 |
| Ownership |  |  |
| Non-profit, n (%) |  | REF |
| For-profit, n (%) |  | -0.07 (-0.16, 0.01); 0.10 |
| Government, n (%) |  | 0.12 (0.05, 0.20); 0.002 |
| Rural Location, n (%) |  | 0.05 (-0.04, 0.14); 0.28 |
| Teaching Hospital, n (%) |  | 0.05 (-0.05, 0.16); 0.31 |
| Nurses Employed by Hospital (in 100s), n (%) |  | 0.00 (-0.00, 0.00); 0.92 |
| Beds in hospital (in 100s) |  | -0.05 (-0.07, -0.03); <0.0001 |
| Total Population in HSA (in 10,000s) |  | -0.003 (-0.004, -0.002); <0.001 |
| Median Age |  | 0.01 (0.001, 0.02); 0.046 |
| African American in HSA, % |  | -0.014 (-0.017, -0.011); <0.0001 |
| Mean income in HSA (in $10,000s) |  | -0.16 (-0.19, -0.13); <0.0001 |
| Poverty in HSA, % |  | -0.016 (-0.024, -0.007); <0.0001 |
| Insurance in HSA, % |  | 0.002 (-0.005, 0.01); 0.52 |
| State dual (Medicaid-Medicare) enrollees (in 10,000s) |  | -0.003 (-0.005, -0.002); <0.0001 |
| Intercept |  | 12.5 (11.7, 13.3); <0.0001 |

**Supplementary Table 2: Results of mixed linear models examining the effect of Medicaid-expansion on 30-day heart failure mortality:** Hospital was used as random intercept and an interaction between Medicaid-expansion (ME) and time was included in the model. The time here is divided by non-overlapping 3-years running average of 30-days heart failure (HF) mortality. Two time period (2005-08 and 2008-11) were before the ME and rest of the three time periods were after ME. The interaction results below show that the three time periods after ME were associated with lower 30-day HF mortality in ME states than non-ME states.

|  | **Unadjusted** | **Adjusted** |
| --- | --- | --- |
| 2005-08 | REF | REF |
| 2008-11 | 0.51 (0.43, 0.59); <0.0001 | 0.51 (0.43, 0.59); <0.0001 |
| 2011-14 | 0.62 (0.54, 0.70); <0.0001 | 0.64 (0.56, 0.73); <0.0001 |
| 2014-17 | 0.72 (0.63, 0.81); <0.0001 | 0.74 (0.65, 0.84); <0.0001 |
| 2017-19 | 0.23 (0.13, 0.33); 0.001 | 0.28 (0.17, 0.38); <0.0001 |
| Non-ME State | REF | REF |
| ME State | -0.12 (-0.22, -0.02); 0.021 | -0.05 (-0.16, 0.05); 0.31 |
| 2008-11 x ME State | -0.05 (-0.15, 0.06); 0.40 | -0.04 (-0.15, 0.07); 0.47 |
| 2011-14 x ME State | -0.16 (-0.27, -0.04); 0.006 | -0.16 (-0.27, -0.04); 0.006 |
| 2014-17 x ME State | -0.15 (-0.28, -0.02); 0.023 | -0.14 (-0.28, -0.01); 0.032 |
| 2017-19 x ME State | -0.20 (-0.34, -0.06); 0.006 | -0.20 (-0.35, -0.06); 0.005 |
| Ownership |  |  |
| Non-profit, n (%) |  | REF |
| For-profit, n (%) |  | -0.08 (-0.16, 0.01); 0.07 |
| Government, n (%) |  | 0.13 (0.05, 0.20); 0.001 |
| Rural Location, n (%) |  | 0.07 (-0.02, 0.16); 0.12 |
| Teaching Hospital, n (%) |  | 0.04 (-0.06, 0.15); 0.43 |
| Nurses Employed by Hospital (in 100s), n (%) |  | -0.000 (-0.001, 0.00); 0.36 |
| Beds in hospital (in 100s) |  | -0.05 (-0.07, -0.03); <0.0001 |
| Total Population in HSA (in 10,000s) |  | -0.003 (-0.003, -0.002); <0.001 |
| Median Age (in years) in HSA |  | 0.01 (0.00, 0.02); 0.04 |
| African American in HSA, % |  | -0.015 (-0.018, -0.012); <0.0001 |
| Mean income in HSA (in $10,000s) |  | -0.16 (-0.19, -0.13); <0.001 |
| Poverty in HSA, % |  | -0.016 (-0.024, -0.008); <0.001 |
| Insurance in HSA, % |  | 0.003 (-0.002, 0.008); 0.22 |
| State dual (Medicaid-Medicare) enrollees (in 10,000s) |  | -0.003 (-0.004, -0.002); <0.001 |
| Intercept |  | 12.0 (11.4, 12.7); <0.0001 |

**Supplementary Table 3:** **Relationship Between Heart Failure Mortality and New Medicaid Enrollees After Medicaid Expansion**- This table displays the unadjusted and adjusted relationship between 30-day risk standardized heart failure mortality and new Medicaid enrollees after Medicaid-expansion using mixed linear models with hospital as random-intercepts.

|  | **Unadjusted** | **Adjusted** |
| --- | --- | --- |
| New Medicaid Enrollees (in 10,000s) | -0.003 (-0.004, -0.002); <0.0001 | -0.002 (-0.003, -0.002); <0.001 |
| Ownership |  |  |
| Non-profit, n (%) |  | REF |
| For-profit, n (%) |  | -0.15 (-0.37, 0.07); 0.17 |
| Government, n (%) |  | 0.30 (0.10, 0.49); 0.003 |
| Teaching Hospital, n (%) |  | 0.06 (-0.12, 0.25); 0.49 |
| Beds in hospital (in 100s) |  | -0.08 (-0.12, -0.05); <0.001 |
| Nurses Employed by Hospital (in 100s), n (%) |  | 0.001 (-0.001, 0.002); 0.73 |
| Rural Location, n (%) |  | 0.03 (0.17, 0.22); <0.001 |
| Total Population in HSA (in 10,000s) |  | -0.003 (-0.005, -0.001); <0.001 |
| Median Age, years |  | 0.02 (0.002, 0.04); 0.03 |
| African American in HSA, % |  | -0.02 (-0.03, -0.01); <0.001 |
| Mean income in HSA (in $10,000s) |  | -0.24 (-0.30, -0.19); <0.001 |
| Poverty in HSA, % |  | -0.02 (-0.04, -0.01); 0.002 |
| Insurance in HSA, % |  | 0.000 (-0.01, 0.01); 0.93 |
